# Association of the serum uric acid to creatinine ratio with metabolic syndrome in the middle age and older population in China

**DOI:** 10.3389/fendo.2022.1060442

**Published:** 2022-12-21

**Authors:** Danrong Zhong, Dongchen Liu, Yongtian Guo, Haoyin Huang, Lu Li, Fangqin Wu, Suli Huang

**Affiliations:** ^1^ Department of Cardiovascular Medicine, The Second Affiliated Hospital of Shantou University Medical College, Shantou, China; ^2^ Department of Neurosurgery, The Second Affiliated Hospital of Shantou University Medical College, Shantou, China; ^3^ Guangdong Provincial Key Laboratory of Infectious Diseases and Molecular Immunopathology, Shantou University Medical College, Shantou, China; ^4^ Department of Cardiovascular Medicine, The Second Affiliated Hospital of Nanchang University, Nanchang, Jiangxi, China; ^5^ Shenzhen Center for Disease Control and Prevention, Shenzhen, China

**Keywords:** serum uric acid-to-creatinine ratio (SUACr), metabolic syndrome, cross-sectional research, population study, multiple statistical analyses

## Abstract

**Background:**

Metabolic syndrome (MetS) has attracted great interest, with an increasing prevalence. Recent studies have shown that the serum uric acid-to-creatinine ratio (SUACr) might be an excellent biomarker for MetS risk prediction in diabetic patients and postmenopausal women. However, the relationship between SUACr and MetS in a middle-aged and older population remains unclear.

**Methods:**

A total of 1277 participants were included in this cross-sectional study. Logistic regression modelling was performed to assess the association between SUACr and MetS in the total population. The dose–response relationship of SUACr and MetS was further assessed by a restricted cubic spline model (RCS). Furthermore, to explore the relationships between the levels of SUACr and the number of metabolic components, analysis of covariance (ANCOVA) was applied.

**Results:**

The levels of SUACr were lower in the non-MetS participants (OR 1.60, 95% CI 1.36 to 1.89; *P*<0.001),. Positive and dose–response relationships were further confirmed by the RCS model. We also found that, with increased number of components, the SUACr tended to increase. Moreover, values of SUACr were strongly related to levels of triglycerides (TGs), body mass index (BMI), blood glucose levels, systolic blood pressure/diastolic blood pressure (SBP/DBP), and hypertension. In addition, the positive association between SUACr and MetS also occurred in those patients with normal uric acid levels.

**Conclusion:**

Elevated values of SUACr were strongly associated with an increased risk of MetS; this positive relationship remained in those individuals with normal uric acid levels.

## Introduction

The metabolic syndrome (MetS) is characterized as a clustering of metabolic disorders, including hypertension, hyperglycemia, overweight/obesity, high levels of triglyceride (TGs), or low levels of high-density lipoprotein cholesterol (HDL-c) (1). The MetS had been proven to be associated with an increased risk of diabetes and cardiovascular mortality and morbidity. MetS patients also exhibited a four times greater risk of fatal coronavirus disease 2019 (COVID-19) outcomes compared with MetS-free participants (2).

Apart from these serious life-threatening conditions, emerging prevalence data have shown that MetS is becoming increasingly common in both economically developing and developed countries. According to a report based on the data from 31 provinces in China, Lu et al. indicated that MetS affects nearly 454 million adults in mainland China, and the prevalence of MetS was 13.3% in those aged 35–64 years (3). In addition, nearly 35% of US adults have suffered from MetS and 50% of those were older than 60 years (4). Greater awareness of MetS and its health burden may have contributed to improvements in optimizing treatment and new biomarkers of this disease given the prevalence rate in the world.

Serum uric acid (SUA) is a major product of purine metabolism (5), and has been shown to be a risk factor for various adverse health outcomes, such as cardiovascular disease, stroke, metabolic syndrome, and metabolic-associated fatty liver disease (6–9). However, conflicting research exists (10–12). The controversial conclusions may be explained by the fact that levels of endogenous uric acid depend on renal clearance function. Consequently, renal function-normalized uric acid (the serum uric acid-to-creatinine ratio, SUACr) has emerged as a new biomarker and is regarded as a superior indicator of net uric acid production (13–16). Several studies have indicated that SUACr is associated with metabolic disorders and is a predictor for total mortality (13, 14). Previous researchers have indicated that SUACr was more sensitive than SUA in relation to MetS in diabetic patients and postmenopausal women (15, 16). To date, the relation between SUACr and MetS among a middle-aged and older population has not been reported, especially the effect on MetS within normal uric acid concentrations. In the present study, we aim to investigate the association between SUACr and MetS and its components in middle-aged and older Chinese people.

## Methods and materials

### Study participants

This cross-sectional research was a retrospective study that was conducted with 1277 patients who were aged between 31 and 91 years. All participants were recruited in the Eighth Affiliated Hospital of Sun Yat-Sen University by routine physical examination between 2012 and 2017. Patients with malignancies or neurological disease, peripheral arterial occlusive disease, and cerebrovascular disease were excluded. All participants were outpatients. Informed consent was obtained from all enrolled participants. The ethics committees of the Shenzhen Center for Disease Control and Prevention approved this research.

### Data collection

A structural questionnaire was applied to collect demographic information including gender, age, smoking and drinking status, history of diseases, and medical treatments. The anthropometric indices were measured by well-trained healthcare workers following standardized protocols. The collected measurements included weight, height, and diastolic and systolic blood pressure. Blood samples were collected and tested for different indexes by laboratory assays.

### Definition of the MetS

According to the Chinese Diabetes Society Criteria (17), the patients were defined as having MetS if they had three or more of the following:

(1) a body mass index (BMI) ≥25 kg/m^2^;
(2) were receiving treatment for hypertension or had a diastolic blood pressure (DBP) ≥90mm/Hg or (and) a systolic blood pressure (SBP) ≥140mm/Hg;
(3) had been diagnosed with diabetes or had a fasting plasma glucose (FBG) concentration ≥6.1mmol/l;
(4) were female and had HDL-c levels <1.0 mmol/l, were male and had a HDL-c concentration <0.9 mmol/l, or/and had TG levels ≥1.7 mmol/l.

### Statistical analyses

Characteristics (from laboratory assessments) and general information of the study participants were described as medians with interquartile ranges (IQRs) or means ± standard deviations (SDs), or frequencies with percentages. Differences between groups were examined using the Wilcoxon rank-sum test or the Student’s *t*-test, or chi-squared test based on the characteristics of the data. Spearman’s rank correlation test was conducted to assess the correlations between SUACr and the components of MetS.

The logistic regression models were used to calculate the odds ratios (ORs) and 95% confidence intervals (95% CIs) for the associations between SUACr and the prevalence of MetS. The covariates adjusted for in the logistic regression model included gender, age, smoking and drinking status, and eGFR. To explore the dose–response relationships between SUACr levels and the risk of MetS, we performed the restricted cubic spline (RCS) with knots at the 10th, 50th and 90th percentiles, and the reference values (OR=1) were set at the 10th percentiles. Furthermore, the difference of SUACr in the participants with a diverse number of MetS components was assessed by the analysis of covariance (ANCOVA), and presented as estimated marginal means with 95% CIs. To evaluate the associations between SUACr and the different combinations of MetS, we classified the components of MetS according to the definition of the disease; multivariable logistic regression was applied. In addition, to evaluate the associations between SUACr levels and the components of MetS, a logistic regression model was applied, the SUACr levels were distributed into quartiles according to the concentrations of the total population, and the covariates were as same as the model 1.

Statistical analyses were conducted using R 3.33 (Lucent Technologies, USA) and SPSS 16.0 (Statistical Package for the Social Science, Chicago, IL, UAS). A two-tailed *p*-value <0.05 was considered to be statistically significant.

## Results

### General information

The general characteristics of the study participants are summarized in **Table 1**. Participants with MetS were older than non-MetS (*P*<0.001), and more men were found in the MetS cohort (*P*=0.003). Participants with MetS presented with higher SBP, DBP and BMI and higher levels of TGs, FBG, uric acid, and SUACr (*P*<0.001) than the non-MetS participants. They also showed lower levels of HDL-c and lower eGFRs than the non-MetS individuals (*P*=0.002, and *P*=0.010, respectively). The prevalence of diabetes or hypertension was both higher in the MetS group (*P*<0.001).

**Table 1:** General characteristics of the study population.

Variables	Total population	Non-MetS (n=1128)	MetS (n=149)	*P*-value
	(n=1277)			
Age, years	59.22 ± 9.47	58.79 ± 9.52	62.51 ± 8.44	<0.001^a^
Male, n (%)	566 (44.30)	483 (42.82)	83 (55.70)	0.003^c^
BMI (kg/m2)	23.76 ± 2.71	23.41 ± 2.57	26.38 ± 2.26	<0.001^a^
SBP, mmHg	129.00 (119.00, 138.00)	127.00 (117.00, 136.00)	141.50 (131.80, 152.30)	<0.001^b^
DBP, mmHg	81.00 (74.00, 88.00)	80.00 (74.00, 87.00)	87 (80.00, 95.00)	<0.001^b^
Hypertension, n (%)	378.00 (29.60)	275.00 (24.38)	103.00 (69.10)	<0.001^c^
Diabetes, n (%)	68.00 (5.30)	37.00 (3.28)	31.00 (2.81)	<0.001^c^
FPG (mmol/l)	5.41 (5.06, 5.88)	5.36 (5.02, 5.74)	6.20 (5.63, 6.67)	<0.001^b^
TC (mmol/l)	5.37 (4.65, 6.08)	5.35 (4.65, 6.05)	5.51 (4.63, 6.34)	0.647^b^
TG (mmol/l)	1.25 (0.87, 1.78)	1.18 (0.84, 1.61)	2.07 (1.73, 2.84)	<0.001^b^
HDL-c (mmol/l)	1.55 (1.39, 1.63)	1.41 (1.18, 1.66)	1.21 (1.02, 1.39)	0.002^b^
LDL-c (mmol/l)	3.07 (2.52, 3.71)	3.07 (2.54, 3.69)	3.08 (2.44, 3.84)	0.975^b^
eGFR (ml/min/1.73m2)	100.02 (85.09, 114.84)	100.67 (86.07, 115.86)	96.92 (78.99, 110.20)	0.010^b^
UA	330 (281, 389)	326 (277, 383)	373 (321, 446)	<0.001^b^
SCr	69 (59, 83)	68 (58, 83)	74 (61, 88)	0.005^b^
SUACr	4.67 (4.02, 5.46)	4.62 (4.00, 5.42)	5.02 (4.36, 5.85)	<0.001^b^

BMI, body mass index; SBP, systolic blood pressure; DBP, diastolic blood pressure; FPG, fasting blood glucose; TC, total cholesterol; TG, triglyceride; HDL-c, high-density lipoprotein cholesterol; LDL-c, low-density lipoprotein cholesterol. SUACr, serum uric acid-to-creatinine ratio.

Data are presented as a number (percentage) for categorical data, mean ± SD for parametrically distributed data or median (interquartile range) for non-parametrically distributed data.

The Student’s t-test or Wilcoxon rank-sum test were used for comparison of the continuous variables according to the data distribution, and the chi-squared test was used for the categorical variables.

a :Student’s t-test;

b :Wilcoxon rank-sum test;

c :chi-squared test.

The correlation between SUACr and MetS components are shown in **Table S1**, the adjusted coefficients (r_s_) ranged from 0.95 to –0.12, as the results indicated that SUACr was positively related to BMI (rs=0.23, *P*<0.001) and levels of TGs (rs=0.21, *P*<0.001). The positive associations were also observed in the correlations of SUACr and SBP, DBP, TGs, and FBG with r_s_ =0.10, 0.14,0.21, and 0.07, respectively (all *P*<0.05). The negative association was observed in the HDL-c and SUACr (rs=–0.12, *P*<0.01).

### Association between SUACr and the prevalence of MetS

As shown in **Table 2**, logistic regression indicated that SUACr was positively associated with the risk of MetS (ORs 1.60, 95% CI 1.36 to 1.89; *P*<0.001) after adjustment for gender, age, smoking, drinking, and eGFR.

**Table 2:** The associations between SUACr and the prevalence of MetS.

Variable	OR (95% CI)	*P*
SUA/Cr		
Crude	1.06 (1.01 to 1.11)	<0.001
Model1	1.60 (1.36 to 1.89)	<0.001

Model 1 was adjusted for age, sex, smoking and drinking status, and eGFR.

To figure out which levels of the SUACr were associated with the prevalence of MetS (**Table 3**), we classified SUACr levels into quartiles based on the distribution of the non-MetS population. Compared with the lowest quartile, the adjusted ORs and 95% CIs were 2.67 (1.50 to 4.74) and 3.86 (2.11 to 7.08) in the third (4.62 to 5.42) and fourth quartiles (≥5.42), respectively.

**Table 3:** Odds ratios (95% confidence intervals) for the MetS associated with quartiles of SUACr.

Variable	Q1	Q2	Q3	Q4	*P-*trend
SUA/Cr	≤4.0	4.0-4.62	4.62-5.42	≥5.42	
Crude	1.00(Reference)	1.46 (0.82 to 2.58)	1.98(1.16 to 3.40)	2.45(1.45 to 4.14)	<0.001
Model1	1.00(Reference)	1.80 (0.99 to 3.24)	2.67 (1.50to 4.74)	3.86(2.11 to 7.08)	<0.001

Levels of SUACr were classified into quartiles based on the distributions in the non-MetS population.

Model 1 was adjusted for age, gender, smoking and drinking status, and eGFR.

**Figure 1:**
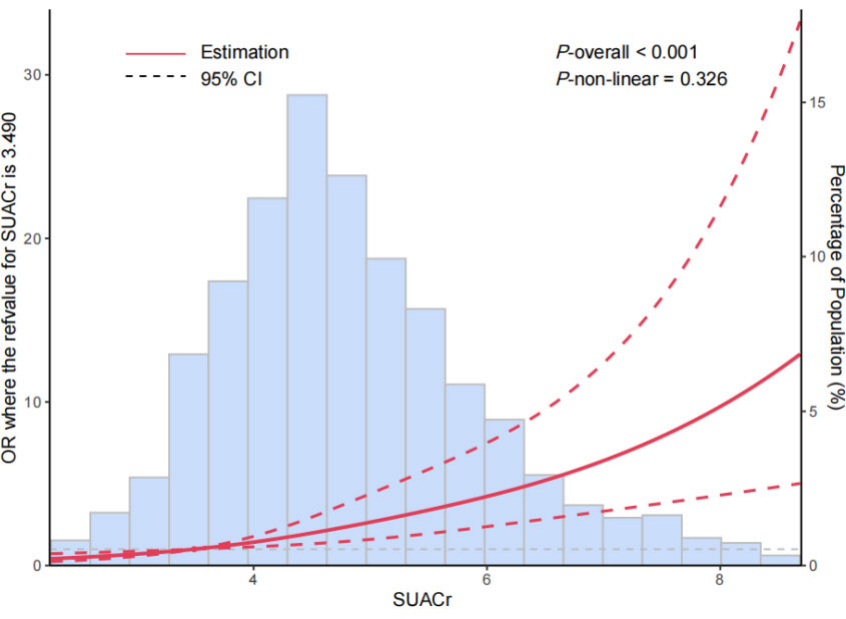
The restricted cubic spline for the relationships between levels of serum uric acid-to-creatinine ratio (SUACr) and the risk of metabolic syndrome (MetS). The adjusted ORs (red lines) and 95% confidence intervals (dashed lines) were calculated based on the restricted cubic spline models for the level of SUACr. The 10th percentiles were set as the reference values, with knots set at 10th, 50^th^, and 90th percentiles of the SUACr. Adjusted confounders were age, gender, smoking and drinking status, and eGFR.

As shown in the **Figure 1**, the dose–response relationship was evaluated in the RCS model. A linear relationship and increasing trend with MetS prevalence were indicated for SUACr, with the overall association with a *P-*value <0.001. The *P-*value for non-linearity was 0.326.

### Associations between SUACr and the MetS’s components

**Figure 2:**
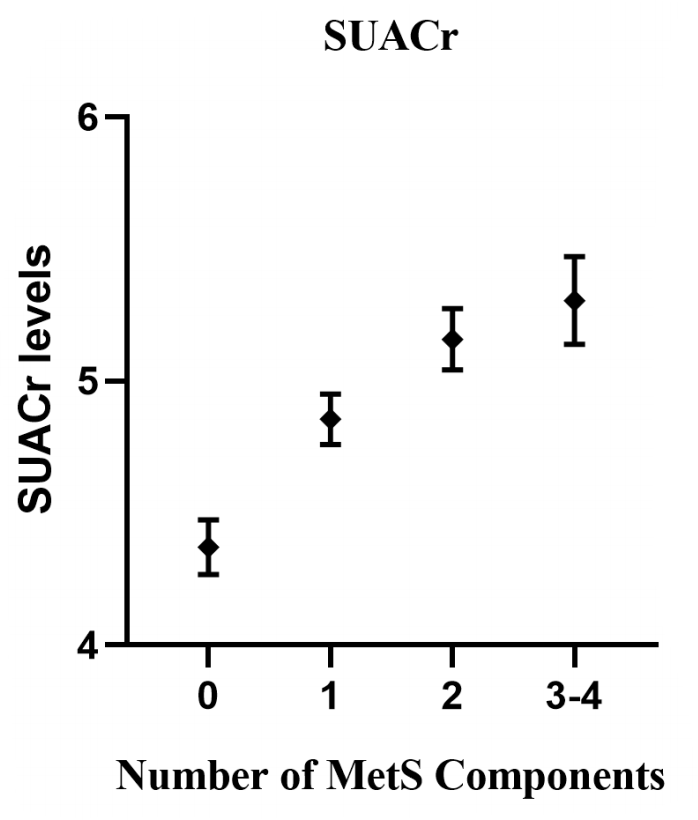
Associations between serum uric acid (SUACr) levels and the number of metabolic syndrome (MetS) components. The analysis of covariance (ANCOVA) model included the levels of SUACr as the dependent variables, and the number of MetS components as a fixed factor. Adjusted confounders were age, gender, smoking and drinking status, and eGFR.

In the ANCOVA analysis (**Figure 2**), with the number of MetS increased, the values of SUACr tended to decrease with the estimated marginal means for the number of 0, 1, 2, 3 or 4 components of MetS, which were 4.37 (95% CI 4.27 to 4.47), 4.86 (95% CI 4.76 to 4.95), 5.16 (95% CI 5.04 to 5.27) and 5.31 (95% CI 5.14 to 5.47), respectively.

Moreover, to analyze the associations between SUACr and different combination of MetS components, we calculated the OR (95% CI) for the whole population using multivariable logistic regression (**Table 4**). The adjusted ORs (95% CIs) were 0.85 (0.55 to 1.31) for BHD, 1.65(1.27 to 2.15) for HTB, 1.83(1.28 to 2.61) for TDB, 1.48(1.11 to1.98) for HDT, and 1.62 (1.14 to 2.31) for HDTB. All *P*-values were <0.05.

**Table 4:** Odds ratios (95% confidence intervals) for the different combination of MetS components associated with SUACr.

Variable	Adjusted OR (95% CI)	*P*
BHD	0.85 (0.55 to 1.31)	0.014
HTB	1.65 (1.27 to 2.15)	<0.001
TDB	1.83 (1.28 to 2.61)	0.001
HDT	1.48 (1.11 to 1.98)	0.007
HDTB	1.62 (1.14 to 2.31)	0.008

the logistic model was adjusted for age, gender, smoking and drinking status, and eGFR.

The MetS phenotype were defined as:

B: BMI≥25 kg/m^2^.

H: SBP≥140mm/Hg/DBP≥90mm/Hg or taking medicine for hypertension.

D: Diagnosed with diabetes or fasting plasma glucose≥6.1mmol/l.

T: HDL-c<1.0 mmol/l in women or HDL-c<0.9 mmol/l in men or/and TG ≥1.7 mmol/l.

We further evaluated the relations of SUACr and the components of MetS (**Table 5**). Results indicated that SUACr was positively associated with BMI (OR 3.08, 95% CI: 2.06 to 4.59) and FBG (OR 1.84, 95% CI 1.12 to 3.04). Such positive relations were also suggested for hypertension and SBP/DBP, with ORs of 2.05 (95% CI 1.36 to 3.09) and 1.96 (95% CI 1.14 to 3.39) in the fourth quartile, respectively. No significant association was found in HDL-c concentrations, but strong increasing trends were found in the levels of TGs as the levels of SUACr increased (OR 6.46, 95% CI 4.21 to 9.92).

**Table 5:** Associations of the SUACr levels with the components of MetS.

Variables	Odd Ratio (95% CI)				*P*-trend
SUACr	Q1 (n=318)	Q2 (n=318)	Q3 (n=317)	Q4 (n=318)	
BMI ≥25kg/m^2^	1.00 (Ref)	1.37 (0.95 to 1.97)	1.75 (1.21 to 2.54)	3.07 (2.06 to 4.57)	<0.001
Glu ≥6.1mmol/l	1.00 (Ref)	1.17 (0.73 to 1.88)	1.45 (0.90 to 2.32)	1.87 (1.14 to 3.08)	0.009
SBP ≥140mmHg/DBP ≥90mmHg	1.00 (Ref)	0.91 (0.58 to 1.43)	1.27 (0.82 to 1.99)	1.72 (1.08 to 2.74)	0.007
Hypertension	1.00 (Ref)	1.12 (0.76 to 1.65)	1.72 (1.17 to 2.53)	2.01 (1.33 to 3.03)	<0.001
Diabetes	1.00 (Ref)	0.68 (0.31 to 1.48)	0.95 (0.46 to 2.00)	1.09 (0.50 to 2.37)	0.629
TG ≥1.7mmol/l	1.00 (Ref)	1.58 (1.05 to 2.36)	2.39 (1.60 to 3.58)	6.41 (4.18 to 9.83)	<0.001
HDL-c ≤0.9 for men, ≤1.0 for women	1.00 (Ref)	1.11 (0.77 to 1.62)	0.96 (0.65 to 1.43)	1.43 (0.96 to 2.15)	0.102

SBP, systolic blood pressure; DBP, diastolic blood pressure; BMI, body mass index; TG, triglyceride; HDL-c, high-density lipoprotein.

A logistic regression model was applied to examine the relationship between SUACr levels and the components of MetS.

Tests for trend were conducted by using the median of each quartile category as a continuous variable, adjusting for age, sex, smoking and drinking status, and eGFR.

### The associations of SUACr and MetS with normal UA levels

In addition, based on the individuals with normal uric acid (UA) levels (SUA<360 umol/l for women and SUA<420 umol/l for men) (18), we found that, in the logistic regression model (**Table S2**), such positive associations remained with an OR of 1.56 (95% CI 1.1 to 2.06) after adjustment for the same covariates of gender, age, smoking, drinking, and eGFR. The same linear associations were still indicated in the RCS model (**Figure S1**), with the overall association having a *P*-value of 0.005 and a *P*-value for non-linearity of 0.675. When we performed a stratified analysis by age in this normal uric acid participants (**Table S3**), we found a positive relationship in the middle-aged participant group with an OR of 2.05 (95% CI 1.28 to 3.28), but such positive relationships were not observed in the older participants with an OR of 1.52 (95% CI 0.84 to 2.73).

## Discussion

In this cross-sectional study, we systematically evaluated the associations of SUACr with the MetS risk in a middle-aged and older population of Chinese people. We found that, related to the first quartile, when SUACr levels were >4.62, SUACr was associated with an increased risk of MetS in a dose–response curve, even after adjusting for multiple risk factors. Notably, positive associations of SUACr and MetS still remained in 961 individuals with normal uric acid levels. Moreover, we found that SUACr was positively related to the components of MetS, namely, TG, BMI, SBP/DBP, hypertension, and FBG.

The role of uric acid in the prevalence of MetS has long been argued. In a cross-sectional analysis, Ford et al. found that serum levels of uric acid are significantly associated with the risk of MetS and its components in US children and adolescents (19). However, other retrospective research failed to demonstrate a strong association between SUA and MetS, and no associations with components of MetS were found (12). In fact, the inconsistencies may be due to renal clearance dysfunction, that is, those individuals with lower eGFRs are more likely to have higher uric acid concentrations (20) and, therefore, renal function-normalized uric acid, will be more precise to reflect net uric acid production (21).

Previous studies have indicated that the SUACr is associated with various diseases. With 778 participants, a cross-sectional study found that SUACr in subjects with non-alcoholic fatty liver disease (NAFLD) was significantly higher than in participants without NAFLD in a South Korean population. This suggested that SUACr may be a reliable marker for predicting NAFLD (22). Such positive relationships between SUACr and NAFLD were also found in a Chinese population and in the US population (21, 23). As an emerging biomarker, SUACr was also found to be related to renal dysfunction (24), that is, a study comprising 185 men and 175 women found that SUACr was independently associated with future renal function decline among diabetic patients (OR 3.15, 95% CI 1.66 to 5.95) (24). Previous research suggested that SUACr is strongly associated with MetS under some specific conditions, such as MetS in diabetic patients or postmenopausal women (15, 16). Consistent with our results, in a retrospective study with 912 Chinese postmenopausal women, Jing et al. suggested that SUACr was an independent risk factor for MetS with an OR of 2.93 (95% CI 2.39 to 3.60) after adjusting for multiple confounders (15). With 332 Saudi type 2 diabetes mellitus participants, Nasser et al. also found that the SUACr in type 2 diabetic patients was strongly associated with full MetS, as well as its individual components (16). In a prospective cohort study, with 1072 community-dwelling persons, Japanese researchers found that baseline SUACr was independently and significantly associated with MetS after the 3-year follow-up and that baseline SUACr was a strong predictor of MetS in women compared with men (14). To our knowledge, the relationship between SUACr and MetS has not yet been fully described in the middle-aged and older population of Chinese people. To address this gap, we performed the present analysis.

The mechanisms that lead to risk of MetS in high SUACr individuals are not completely understood. Recent studies have reported that higher SUA may be related to MetS components. A cross-sectional study had documented that hyperuricemia is related to dyslipidemia and adiposity in 14,618 northwestern Chinese people (25). The lipid metabolic disorder and uric acid are mutually related. Elevated levels of uric acid can lead to a decline in lipoprotein enzymes that impact lipid metabolism, and, ultimately, leads to changes in the adipokines that regulate fat synthesis (26). The unfavorable effect on blood pressure has been confirmed by previous research (27), uric acid can activate the renin–angiotensin system that can be activated by uric acid, thus decreasing renal blood flow, inducing endothelial cell dysfunction, and stimulating vascular smooth muscle cell proliferation in systemic circulation. All the above mechanisms can be explained the occurrence of hypertension (28, 29). As for the positive relation of FBG, consistent with our finding, a prospective analysis of 4883 participants from the Framingham Heart Study and a cohort study recruiting 4292 patients, indicated that individuals with hyperuricemia developed diabetes at a significantly higher rate than those with normal uric acid levels (30). The effect of uric acid on blood glucose may explain by the fact that uric acid amplifies oxidative stress in adipocytes by increasing monocyte chemotactic protein-1 and decreasing adiponectin (31). The pro-oxidative action may accelerate adipose formation (32, 33) and contribute to insulin resistance (34). Consistent with our cross-sectional research, we found that SUACr was positively related to the prevalence of MetS and the components of the disease, specifically, TG, BMI, SBP/DBP, hypertension, and FBG.

Our research had some strengths, the present cross-sectional study was carried out with a large sample size, and all the participants were enrolled from the general population. Multiple statistical methods were conducted to systematically evaluate the relationship between SUACr and MetS. However, several limitations should be acknowledged. First, the cross-sectional study hid the causal association between SUACr and the risk of MetS. Second, the information needed to decrease the confounding influences, such as diet and physical exercise, should be collected in any future analysis. Finally, our findings were based on the data from only a fraction of the participants who had undergone health examinations; thus, our results might not be generalizable to all Chinese individuals.

## Conclusion

The present research showed that higher levels of SUACr levels might increase the risk of MetS and its components in a dose–response relationship in middle-aged and older Chinese people. This finding indicates that we should pay more attention to high SUACr levels due to its positive relationship to MetS risk. Lowering SUACr levels might be a useful strategy for clinical doctors to diagnose MetS patients and to reduce the health and economic burden caused by MetS. Further analysis is required to determine the causal relationship of SUACr in the pathogenesis of MetS.

## Data availability statement

The raw data supporting the conclusions of this article will be made available by the authors, without undue reservation.

## Ethics statement

The ethics committees of the Shenzhen Center for Disease Control and Prevention approved this research (SZCDC[2019]No.006A). The patients/participants provided their written informed consent to participate in this study.

## Author contributions

DZ: Conceptualization, Methodology, Formal analysis, Investigation, Data curation, Writing – original draft, Writing – review, and editing; DL: Conceptualization, Methodology, Formal analysis, Writing – review and editing; YG: Conceptualization, Writing – review and editing; HH: Methodology; LL: Conceptualization, Methodology, Investigation, Writing – review, and editing, Funding acquisition. FW: Conceptualization, Methodology, Investigation, Formal analysis, Writing – review, and editing. SH: Conceptualization, Methodology, Writing – review and editing, Funding acquisition. All authors contributed to the article and approved the submitted version.

## References

[1] Alberti KG Zimmet P Shaw J Group IDFETFC. The metabolic syndrome–a new worldwide definition. Lancet (2005) 366(9491):1059–62. doi: 10.1016/S0140-6736(05)6702-8 16182882

[2] Xie J Zu Y Alkhatib A Pham TT Gill F Jang A. Metabolic syndrome and COVID-19 mortality among adult black patients in new Orleans. Diabetes Care (2020) 44(1):188–93. doi: 10.2337/dc20-1714 PMC7783937 32843337

[3] Lu J Wang L Li M Xu Y Jiang Y Wang W. Metabolic syndrome among adults in China: The 2010 China noncommunicable disease surveillance. J Clin Endocrinol Metab (2017) 102(2):507–15. doi: 10.1210/jc.2016-2477 27898293

[4] Aguilar M Bhuket T Torres S Liu B Wong RJ. Prevalence of the metabolic syndrome in the united states, 2003-2012. Jama (2015) 313(19):1973–4. doi: 10.1001/jama.2015.4260 25988468

[5] Cho SK Chang Y Kim I Ryu S. U-Shaped association between serum uric acid level and risk of mortality: A cohort study. Arthritis Rheumatol (2018) 70(7):1122–32. doi: 10.1002/art.40472 29694719

[6] Chen JH Chuang SY Chen HJ Yeh WT Pan WH. Serum uric acid level as an independent risk factor for all-cause, cardiovascular, and ischemic strok e mortality: a Chinese cohort study. Arthritis Rheum (2009) 61(2):225–32. doi: 10.1002/art.24164 19177541

[7] Chen YL Li H Li S Xu Z Tian S Wu J. Prevalence of and risk factors for metabolic associated fatty liver disease in an urban population in China: a cross-sectional comparative study. BMC Gastroenterol (2021) 21(1):212. doi: 10.1186/s12876-021-01782-w 33971822 PMC8111711

[8] Fang J Alderman MH. Serum uric acid and cardiovascular mortality the NHANES I epidemiologic follow-up study, 1971-1992. n ational health and nutrition examination survey. Jama (2000) 283(18):2404–10. doi: 10.1001/jama.283.18.2404 10815083

[9] Ishizaka N Ishizaka Y Toda E Nagai R Yamakado M. Association between serum uric acid, metabolic syndrome, and carotid atherosclerosis in Japanese indi viduals. Arterioscler Thromb Vasc Biol (2005) 25(5):1038–44. doi: 10.1161/01.ATV.0000161274.87407.26 15746438

[10] Ong G Davis WA Davis TM. Serum uric acid does not predict cardiovascular or all-cause mortality in type 2 diabetes: the freman tle diabetes study. Diabetologia (2010) 53(7):1288–94. doi: 10.1007/s00125-010-1735-7 20349345

[11] Wang Z Lin Y Liu Y Chen Y Wang B Li C. Serum uric acid levels and outcomes after acute ischemic stroke. Mol Neurobiol (2016) 53(3):1753–9. doi: 10.1007/s12035-015-9134-1 25744569

[12] Li L Song Q Yang X. Lack of associations between elevated serum uric acid and components of metabolic syndrome such as hy pertension, dyslipidemia, and T2DM in overweight and obese Chinese adults. J Diabetes Res (2019) 2019:3175418. doi: 10.1155/2019/3175418 31871945 PMC6913180

[13] Moriyama K. The association between the serum uric acid to creatinine ratio and metabolic syndrome, liver functio n, and alcohol intake in healthy Japanese subjects. Metab Syndr Relat Disord (2019) 17(7):380–7. doi: 10.1089/met.2019.0024 31237480

[14] Kawamoto R Ninomiya D Akase T Kikuchi A Kasai Y Kusunoki T. Serum uric acid to creatinine ratio independently predicts incident metabolic syndrome among communit y-dwelling persons. Metab Syndr Relat Disord (2019) 17(2):81–9. doi: 10.1089/met.2018.0055 30614758

[15] Tao J Shen X Li J Cha E Gu PP Liu J. Serum uric acid to creatinine ratio and metabolic syndrome in postmenopausal Chinese women. Med (Baltimore) (2020) 99(17):e19959. doi: 10.1097/MD.0000000000019959 PMC7220721 32332681

[16] Al-Daghri NM Al-Attas OS Wani K Sabico S Alokail MS. Serum uric acid to creatinine ratio and risk of metabolic syndrome in Saudi type 2 diabetic patients. Sci Rep (2017) 7(1):12104. doi: 10.1038/s41598-017-12085-0 28935934 PMC5608718

[17] Pang C Jia L Hou X Gao X Liu W Bao Y. The significance of screening for microvascular diseases in Chinese community-based subjects with var ious metabolic abnormalities. PloS One (2014) 9(5):e97928. doi: 10.1371/journal.pone.0097928 24835219 PMC4023981

[18] Kuo CC Weaver V Fadrowski JJ Lin YS Guallar E Navas-Acien A. Arsenic exposure, hyperuricemia, and gout in US adults. Environ Int (2015) 76:32–40. doi: 10.1016/j.envint.2014.11.015 25499256

[19] Ford ES Li C Cook S Choi HK. Serum concentrations of uric acid and the metabolic syndrome among US children and adolescents. Circulation (2007) 115(19):2526–32. doi: 10.1161/CIRCULATIONAHA.106.657627 17470699

[20] Johnson RJ Bakris GL Borghi C Chonchol MB Feldman D Lanaspa MA. Hyperuricemia, acute and chronic kidney disease, hypertension, and cardiovascular disease: Report of a scientific workshop organized by the national kidney foundation. Am J Kidney Dis (2018) 71(6):851–65. doi: 10.1053/j.ajkd.2017.12.009 PMC7286363 29496260

[21] Ma C Liu Y He S Zeng J Li P Ma C. C-peptide: A mediator of the association between serum uric acid to creatinine ratio and non-alcoholi c fatty liver disease in a Chinese population with normal serum uric acid levels. Front Endocrinol (Lausanne) (2020) 11:600472. doi: 10.3389/fendo.2020.600472 33329401 PMC7711154

[22] Seo YB Han AL. Association of the serum uric acid-to-Creatinine ratio with nonalcoholic fatty liver disease diagnosed by computed tomography. Metab Syndr Relat Disord (2021) 19(2):70–5. doi: 10.1089/met.2020.0086 33314991

[23] Sookoian S Pirola CJ. The serum uric acid/creatinine ratio is associated with nonalcoholic fatty liver disease in the general population. J Physiol Biochem (2022). doi: 10.1007/s13105-022-00893-6 35546386

[24] Kawamoto R Ninomiya D Kikuchi A Akase T Kasai Y Ohtsuka N. Serum uric acid to creatinine ratio is a useful predictor of renal dysfunction among diabetic persons. Diabetes Metab Syndr (2019) 13(3):1851–6. doi: 10.1016/j.dsx.2019.04.023 31235105

[25] Liu F Du GL Song N Ma YT Li XM Gao XM. Hyperuricemia and its association with adiposity and dyslipidemia in Northwest China: results from ca rdiovascular risk survey in xinjiang (CRS 2008-2012). Lipids Health Dis (2020) 19(1):58. doi: 10.1186/s12944-020-01211-z 32238146 PMC7115071

[26] Wang H Sun Y Wang S Qian H Jia P Chen Y. Body adiposity index, lipid accumulation product, and cardiometabolic index reveal the contribution o f adiposity phenotypes in the risk of hyperuricemia among Chinese rural population. Clin Rheumatol (2018) 37(8):2221–31. doi: 10.1007/s10067-018-4143-x 29770928

[27] Cannon PJ Stason WB Demartini FE Sommers SC Laragh JH. Hyperuricemia in primary and renal hypertension. N Engl J Med (1966) 275(9):457–64. doi: 10.1056/NEJM196609012750902 5917940

[28] Choi YJ Yoon Y Lee KY Hien TT Kang KW Kim KC. Uric acid induces endothelial dysfunction by vascular insulin resistance associated with the impairme nt of nitric oxide synthesis. FASEB J (2014) 28(7):3197–204. doi: 10.1096/fj.13-247148 24652948

[29] Ma H Wang X Guo X Li X Qi L Li Y. Distinct uric acid trajectories are associated with different risks of incident hypertension in middl e-aged adults. Mayo Clin Proc (2019) 94(4):611–9. doi: 10.1016/j.mayocp.2018.08.042 30947831

[30] Rathmann W Funkhouser E Dyer AR Roseman JM. Relations of hyperuricemia with the various components of the insulin resistance syndrome in young bl ack and white adults: the CARDIA study. coronary artery risk development in young adults. Ann Epidemiol (1998) 8(4):250–61. doi: 10.1016/s1047-2797(97)002044 9590604

[31] Baldwin W McRae S Marek G Wymer D Pannu V Baylis C. Hyperuricemia as a mediator of the proinflammatory endocrine imbalance in the adipose tissue in a mur ine model of the metabolic syndrome. Diabetes (2011) 60(4):1258–69. doi: 10.2337/db10-0916 PMC3064099 21346177

[32] Johnson RJ Lanaspa MA Gaucher EA. Uric acid: a danger signal from the RNA world that may have a role in the epidemic of obesity, metabo lic syndrome, and cardiorenal disease: evolutionary considerations. Semin Nephrol (2011) 31(5):394–9. doi: 10.1016/j.semnephrol.2011.08.002 PMC3203212 22000645

[33] Lee H Lee YJ Choi H Ko EH Kim JW. Reactive oxygen species facilitate adipocyte differentiation by accelerating mitotic clonal expansion. J Biol Chem (2009) 284(16):10601–9. doi: 10.1074/jbc.M808742200 PMC2667747 19237544

[34] Furukawa S Fujita T Shimabukuro M Iwaki M Yamada Y Nakajima Y. Increased oxidative stress in obesity and its impact on metabolic syndrome. J Clin Invest (2004) 114(12):1752–61. doi: 10.1172/JCI21625 PMC535065 15599400

