# Supplementary material for: Association of the serum uric acid to creatinine ratio with metabolic syndrome in the middle age and older population in China
Source: Front Endocrinol (Lausanne). 2022 Dec 21;13:1060442. doi: 10.3389/fendo.2022.1060442 (PMC9810746; doi:10.3389/fendo.2022.1060442)
Supplement: Supplementary file 1 [file DataSheet_1.doc]

### Supplemental Material

**Association of the serum uric acid to serum creatinine ratio with metabolic syndrome in the middle age and older population in China**

Danrong Zhong^1^, Dongchen Liu^1^, Yongtian Guo^2^, Haoyin Huang^1^, Lu Li^1, 3*^, Fangqin Wu^4*^, Suli Huang^5*^

^1^ *Department of Cardiovascular Medicine, the Second Affiliated Hospital of Shantou University Medical College, Shantou 515000, China*

^2^*Department of Neurosurgery, the Second Affiliated Hospital of Shantou University Medical College, Shantou 515000, China*

*^3^Guangdong Provincial Key Laboratory of Infectious Diseases and Molecular Immunopathology, Shantou University Medical College, Shantou 515041;*

*^4^ Department of Cardiovascular Medicine, The Second Affiliated Hospital of Nanchang University, Jiangxi, 330008, China*

*^5^ Shenzhen Center for Disease Control and Prevention, Shenzhen, 518055, China*

*Address correspondence to

Lu Li, Department of Cardiovascular Medicine, the Second Affiliated Hospital of Shantou University Medical College, Shantou 515000, China. E-mail address: [lilu@stu.edu.cn](mailto:lilu@stu.edu.cn)

Fangqin Wu, Department of Cardiovascular Medicine, The Second Affiliated Hospital of Nanchang University, Jiangxi, China. E-mail address: [wufangqinnice@sina.com](mailto:wufangqinnice@sina.com)

Suli Huang, Shenzhen Center for Disease Control and Prevention, Shenzhen, 518055, China. E-mail address: [huangsuli420@163.com](mailto:huangsuli420@163.com)

**Table of contents**

**Table S1** The correlation between SUACr and the components of MetS (n=1277)

**Table S2** Associations between SUACr and prevalence of MetS in normal uric acid level population (n=961)

**Table S3 The stratified analysis by age in the participants with normal uric acid population**

**Fig .S1** The restricted cubic spline for the relationships between levels of SUACr and the risk of MetS in normal uric acid population. (n=961)

**Table S1 The correlation between SUACr and the components of MetS**

| Variables | rs | *P* | rs’ | *P*’ |
| --- | --- | --- | --- | --- |
| BMI | 0.13 | <0.01 | 0.23 | <0.01 |
| SBP | 0.09 | <0.01 | 0.10 | <0.01 |
| DBP | 0.01 | >0.05 | 0.14 | <0.01 |
| TG | 0.22 | <0.01 | 0.21 | <0.01 |
| HDL-c | -0.06 | <0.05 | -0.12 | <0.01 |
| FBG | 0.06 | >0.05 | 0.07 | 0.02 |
| UA | 0.50 | <0.01 | 0.95 | <0.01 |
| SCr | -0.45 | <0.01 | -0.13 | <0.01 |

Note: BMI, Body Mass Index; SBP, systolic blood pressure; DBP, diastolic blood pressure; TG, triglyceride; HDL-c, high-density lipoprotein cholesterol; FPG, fating blood glucose; UA, Uric Acid; SCr, Serum Creatinine; SUACr, serum uric acid to creatinine ratio.

rs’ and *P*’ were adjusted with age, gender, smoking, drinking status and eGFR.

**Table S2 Associations between SUACr and prevalence of MetS in normal uric acid level population**

| Variable | OR(95%CI) | *P* |
| --- | --- | --- |
| SUA/Cr |  |  |
| Crude | 1.25 (1.00, 1.56) | 0.060 |
| Model1 | 1.56 (1.18, 2.06) | 0.002 |

Note: Model 1, adjusted with age, sex, smoking , drinking status and eGFR.

**Table S3 The stratified analysis by age in the participants with normal uric acid population**

| Variable | OR (95%CI) | *P* |
| --- | --- | --- |
| SUA/Cr |  |  |
| Middle-aged (age between 31-64) | 2.05 (1.28, 3.28) | 0.003 |
| Older participants (age≥65) | 1.52 (0.84, 2.73) | 0.165 |

Note: Model was adjusted with age, sex, smoking, drinking status and eGFR


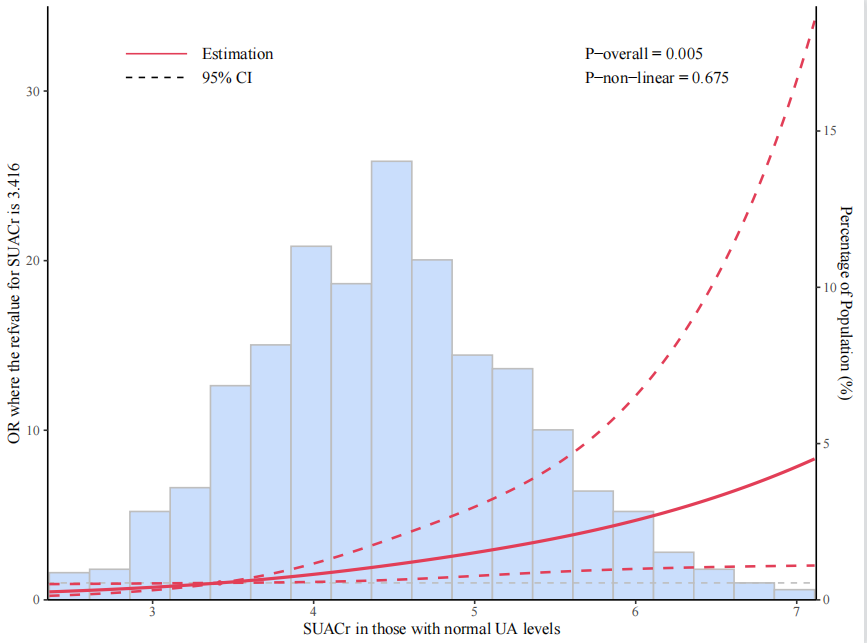


**Fig.S1** **The restricted cubic spline for the relationships between levels of serum uric acid to creatinine ratio (SUACr) and the risk of metabolic syndrome (MetS) in normal uric acid population.**

The adjusted ORs (red lines) and 95% confidence intervals (dashed lines) were calculated based on the restricted cubic spline models for the level of SUACr.

The 10th percentiles were set as the reference values, with knots set at 10th, 50th and 90th percentiles of the SUACr. Adjusted confounders were age, gender, smoking and drinking status, eGFR.
